# Editorial for “Information and Communication Technology-based Assessment for Children with Developmental Needs: Kids Brain Balancer”

**DOI:** 10.31662/jmaj.2024-0215

**Published:** 2024-09-20

**Authors:** Tatsuya Koeda

**Affiliations:** 1 Department of Pediatrics, Tottori Prefectural Rehabilitation Center for Children with Disabilities, Tottori, Japan

**Keywords:** Cognitive function assessment, ICT, Children with disabilities, Special needs education

The purpose of this study was to verify the reliability of a cognitive function assessment using ICT called the Kids Brain Balancer, which is developed as a useful tool for the educational guidance of children with special needs ^(1)^. The authors state that the widely used intelligence test WISC, requiring a qualified psychologist to administer and taking over an hour to complete, also necessitates intervals of over a year between tests. They argue that this is inconvenient for continuously assessing the actual effectiveness of special education programs.

To improve this inconvenience, the authors have developed the Kids Brain Balancer, an assessment that uses ICT to quickly and easily examine cognitive abilities, without requiring special qualifications. As a preliminary study, validation of its reliability and validity has already been completed for adults with cognitive disabilities and high brain dysfunction, thereby positioning this study as its pediatric version ^(2)^. In the field of special needs education, the development of cognitive function tests that can easily and repeatedly assess cognitive abilities using ICT is extremely valuable. No other comparable testing methods are available in Japan, and we look forward to further advancements in this area.

This study concludes that the reliability of the assessment is confirmed through the consistency of the test-retest results, and its validity is validated through correlation with the standard intelligence test (WISC-IV). The following are a few points of concern:

・The assessment comprises 13 subtests. However, the validation in this study covers only nine of these subtests. This study does not mention or discuss the remaining four subtests that were omitted. This raises doubts about the completeness of the overall validation of the Kids Brain Balancer.

・Furthermore, in terms of test-retest reliability, the scores tended to increase in subsequent administrations (second and third) compared with the first, particularly in subtests, such as Drag Race, Follow the Order, and Matching Words. This suggests a potential learning effect from repeated administrations of the Kids Brain Balancer that should be carefully considered.

To pave the way for future developmental advancements, addressing several remaining challenges is imperative, as outlined below. Currently, intellectual structures are often explained using the Cattell-Horn-Carroll (CHC) theory ^(3)^. The latest intelligence test in Japan since last year, the WISC-V, is based on the CHC theory. For the Kids Brain Balancer to gain wide recognition and acceptance as a reliable assessment tool, explicitly outlining the cognitive structural theory that underpins it is crucial.

The Kids Brain Balancer comprises 13 subtests, and the reasons for selecting these 13 subtests need to be explained from the perspective of cognitive function structure theory. It is essential to demonstrate that by administering all 13 subtests, the Kids Brain Balancer provides comprehensive guidance for educating children receiving special education, without deficiencies. This clarification will be instrumental in ensuring the Kids Brain Balancer’s broad adoption and effectiveness in educational settings.

Furthermore, we hope to see examples of how the Kids Brain Balancer can be used for children receiving special need education, categorized by their specific conditions.

## Article Information

### Conflicts of Interest

None
